# Analytical Verification and Method Comparison of the Atellica HEMA 580 Hematology Analyzer for Routine Clinical Laboratory Implementation in a Tertiary Care Setting

**DOI:** 10.7759/cureus.110761

**Published:** 2026-06-12

**Authors:** Mohamed Al Zaabi, Amira Al Mawali, Shaima Al Mamari, Maryam Al Buraiki, Melwin Deepak

**Affiliations:** 1 Department of Hematology, Khoula Hospital, Muscat, OMN

**Keywords:** analytical validation, atellica hema 580, complete blood count, hematology analyzer, method comparison

## Abstract

Accurate complete blood count (CBC) measurements are fundamental to modern hematology practice, and the introduction of new automated hematology analyzers requires rigorous analytical validation to ensure reliability, comparability, and clinical safety. The Atellica HEMA 580 (Siemens Healthineers AG, Erlangen, Germany) is a high-throughput hematology analyzer for which independent Clinical and Laboratory Standards Institute (CLSI)-guided validation data remain limited. This study aimed to perform a comprehensive analytical validation of the Atellica HEMA 580 and to evaluate its analytical comparability with the Sysmex XN-3100 (Sysmex Corporation, Kobe, Japan).

Analytical validation was conducted in accordance with CLSI guidelines. Precision was assessed following CLSI EP05-A3, linearity according to EP06-A, carryover using EP07-A2, and method comparison per EP09-A3. Precision was evaluated using 15 within-run and 15 inter-assay replicates across three quality control levels. Linearity was evaluated using proportional dilution from neat to 1:16. Forty-four paired routine clinical samples were analyzed in parallel on both analyzers using ordinary least squares, Passing-Bablok, and Deming regression, supplemented by Bland-Altman analysis.

Within-run analytical imprecision was low, with coefficients of variation of 0.76% for white blood cells, 0.73% for red blood cells, 0.25% for hemoglobin, and 3.59% for platelets, together with stable inter-assay reproducibility. Linearity of red blood cell and hemoglobin measurements demonstrated high proportionality across the evaluated analytical ranges. Platelet method comparison demonstrated a modest negative proportional bias (Deming slope 0.95; 95% confidence interval, 0.91-0.99). High correlation coefficients across major hematological parameters further supported strong analytical agreement between the two analyzers. Bland-Altman analysis showed minimal systematic bias, and carryover remained low across all parameters.

Atellica HEMA 580 met CLSI analytical validation criteria and demonstrated analytical agreement with the Sysmex XN platform, supporting its use in routine and high-throughput hematology laboratories.

## Introduction

The complete blood count (CBC) is among the most frequently requested laboratory investigations worldwide and plays a central role in diagnostic evaluation, disease monitoring, and therapeutic decision-making across a broad spectrum of clinical conditions [1,2]. CBC parameters are relied upon for both acute clinical assessment and longitudinal patient monitoring, including anemia classification, infection evaluation, chemotherapy follow-up, transfusion support, and critical care decision-making [1]. Given this importance, analytical reliability is essential, as even small systematic or random errors may translate into clinically meaningful consequences, particularly when results are trended over time or interpreted near decision thresholds [2].

As laboratory medicine continues to evolve toward higher throughput, increased automation, and integrated laboratory networks, the introduction of new hematology analyzers requires careful analytical validation prior to clinical implementation [3,4]. Such validation ensures that reported results are precise, accurate, linear across clinically relevant ranges, free from significant carryover, and comparable with existing platforms [3-6]. This is particularly important in laboratories operating multiple analyzer types or transitioning between platforms, where analytical discrepancies may confound clinical interpretation and disrupt continuity of patient data [2,7].

The Clinical and Laboratory Standards Institute (CLSI) provides a robust and widely accepted framework for analytical validation, encompassing precision (EP05), linearity (EP06), carryover (EP07), and method comparison (EP09) [3-6]. These guidelines form the foundation of quantitative analytical evaluation in laboratory medicine and are endorsed by accreditation bodies and peer-reviewed journals [3,4]. Complementary guidance from the International Council for Standardization in Haematology (ICSH) further addresses hematology-specific considerations, including platelet counting methodologies and analytical limitations inherent to cellular measurements [7].

The Atellica HEMA 580 (Siemens Healthineers AG, Erlangen, Germany) is a modern, high-throughput hematology analyzer designed for routine and high-volume clinical laboratories [8]. It employs impedance-based enumeration for leukocytes, erythrocytes, and platelets; cyanide-free photometric hemoglobin measurement; and fluorescence-assisted flow cytometry for reticulocyte analysis. While manufacturer-provided data suggest robust analytical characteristics, independent CLSI-guided validation studies remain limited in the peer-reviewed literature [8].

In contrast, the Sysmex XN-series (Sysmex Corporation, Kobe, Japan) has been extensively evaluated and is widely regarded as a benchmark platform for automated hematology analysis [9-11]. Comparative evaluation against the Sysmex XN-3100 therefore provides a clinically relevant reference for assessing analytical agreement and potential interchangeability. The aim of this study was to perform a CLSI-guided analytical validation of the Atellica HEMA 580 and to evaluate its analytical comparability with the Sysmex XN-3100 across key CBC parameters, including WBC, RBC, hemoglobin, and platelet measurements, to determine its suitability for routine clinical laboratory implementation.

## Materials and methods

Sample handling

Residual adult venous blood samples collected in dipotassium ethylenediaminetetraacetic acid (K_2_EDTA) tubes were used for all analyses. Samples were analyzed within four hours of phlebotomy to minimize pre-analytical variability related to cellular degradation, platelet activation, or leukocyte morphological changes [12]. Samples exhibiting visible clotting, significant hemolysis, or insufficient volume were excluded in accordance with standard laboratory pre-analytical quality criteria [12,13]. All samples represented routine clinical submissions and encompassed a broad analytical range for each evaluated parameter, reflecting the typical case mix encountered in daily laboratory practice.

Instrumentation

The Atellica HEMA 580 employs impedance-based particle counting for WBC, RBC, and platelet enumeration, cyanide-free optical spectrophotometry for hemoglobin measurement, and fluorescence-assisted flow cytometry for reticulocyte analysis [8]. The Sysmex XN-3100, which integrates impedance, optical, and fluorescence measurement principles and has been extensively validated in the literature, served as the comparator analyzer [9-11]. Both analyzers were operated according to manufacturer recommendations, with routine daily quality control and scheduled preventive maintenance performed throughout the study period.

Precision

Analytical precision was assessed in accordance with CLSI EP05-A3 using 15 within-run replicates and 15 inter-assay replicates across three levels of commercial quality control material, with inter-assay testing performed across multiple analytical runs [3]. Coefficients of variation (CVs) were calculated for each parameter. Observed CVs were interpreted in the context of CLSI recommendations and biological variation-derived analytical performance goals.

Linearity

Linearity of RBC and hemoglobin measurements was evaluated using serial proportional dilutions from neat to 1:16 [5]. Observed values were compared with expected concentrations using least-squares regression, residual analysis, and percentage recovery calculations. Linearity of WBC and platelets was not assessed, consistent with CLSI EP06 and ICSH guidance regarding dilution-related cellular artifacts.

Carryover

Carryover was evaluated using standardized high-low testing sequences as specified in CLSI EP07-A2. Carryover percentages were calculated using the CLSI-recommended equation, with values below 1% considered analytically acceptable for routine hematology testing [6].

Method comparison and statistical analysis

Forty-four paired clinical samples spanning a wide analytical range were analyzed in parallel on both analyzers for WBC, RBC, hemoglobin, and platelet measurements. Agreement was assessed using ordinary least squares regression, Passing-Bablok regression, and Deming regression to account for measurement error in both methods [14]. Ninety-five percent confidence intervals (95% CI) were calculated for regression slopes and intercepts to evaluate proportional and constant bias [4,15]. Bland-Altman analysis was performed to assess systematic bias and limits of agreement across the analytical range [16]. Statistical analyses were aligned with CLSI EP09-A3 principles, emphasizing estimation of analytical agreement rather than hypothesis testing. No p-values were calculated, as statistical significance testing is not recommended for analytical validation where clinical relevance is the primary interpretive framework [4].

## Results

Analytical precision

The Atellica HEMA 580 demonstrated low within-run imprecision across all evaluated CBC parameters. Within-run CVs were 0.76% for WBC, 0.73% for RBC, and 0.25% for hemoglobin, all substantially below 1% and exceeding desirable biological variation-derived analytical performance goals. These findings indicate high analytical repeatability and support reliable detection of small but clinically meaningful changes during longitudinal monitoring (Table 1).

**Table 1:** Analytical precision and carryover performance of Atellica HEMA 580 Within-run and between-run precision were assessed according to CLSI EP05-A3 using three levels of commercial quality control (QC) material. Carryover was evaluated following the CLSI EP07-A2 high-low sequence methodology. Results are expressed as coefficients of variation (CV%) or percentage carryover. CLSI: Clinical and Laboratory Standards Institute

Parameter	QC Level	Within-run CV (%)	Between-run CV (%)	Carryover (%)
WBC (×10^9^/L)	Low	0.76	1.02	0.05
	Normal	0.69	0.95	0.04
	High	0.72	0.98	0.06
RBC (×10^12^/L)	Low	0.73	0.88	0.03
	Normal	0.65	0.81	0.02
	High	0.70	0.85	0.04
Hemoglobin (g/dL)	Low	0.25	0.42	0.00
	Normal	0.22	0.39	0.01
	High	0.24	0.41	0.01
Platelets (×10^9^/L)	Low	3.59	4.21	0.11
	Normal	3.12	3.85	0.09
	High	3.40	4.05	0.10

Inter-assay precision across three quality control levels showed minimal day-to-day variability, confirming analytical stability under routine operating conditions. Platelet precision demonstrated higher CVs (3.59%), consistent with known biological and methodological variability of impedance-based platelet enumeration. Across all quality control levels, CVs remained stable with no evidence of drift over the study period.

Carryover

Carryover values ranged from 0.00% to 0.11% across evaluated parameters, remaining well below CLSI EP07-A2 acceptance thresholds and indicating minimal risk of sample-to-sample contamination during high-throughput operation. No parameter demonstrated systematic elevation following high-concentration samples (Table 1).

Linearity

Linearity assessment demonstrated high proportionality for both RBC and hemoglobin measurements across the evaluated dilution ranges. Regression analysis yielded R^2^ values of 0.9998 for RBC and 1.000 for hemoglobin, with recovery values remaining within ±10% of expected concentrations. Residual analysis showed no evidence of non-linearity across the analytical range (Table 2).

**Table 2:** Linearity assessment of RBC and hemoglobin measurements Linearity was evaluated according to CLSI EP06-A using serial proportional dilutions (neat to 1:16). Expected and observed values were compared by least-squares regression and percentage recovery. CLSI: Clinical and Laboratory Standards Institute

Parameter	Analytical range tested	R^2^	Mean recovery (%)	Recovery range (%)
RBC (×10^12^/L)	0.8-7.2	0.9998	100.6	97.8-103.2
Hemoglobin (g/dL)	2.5-22.0	1.000	100.2	98.5-102.1

Method comparison

**Figure 1:**
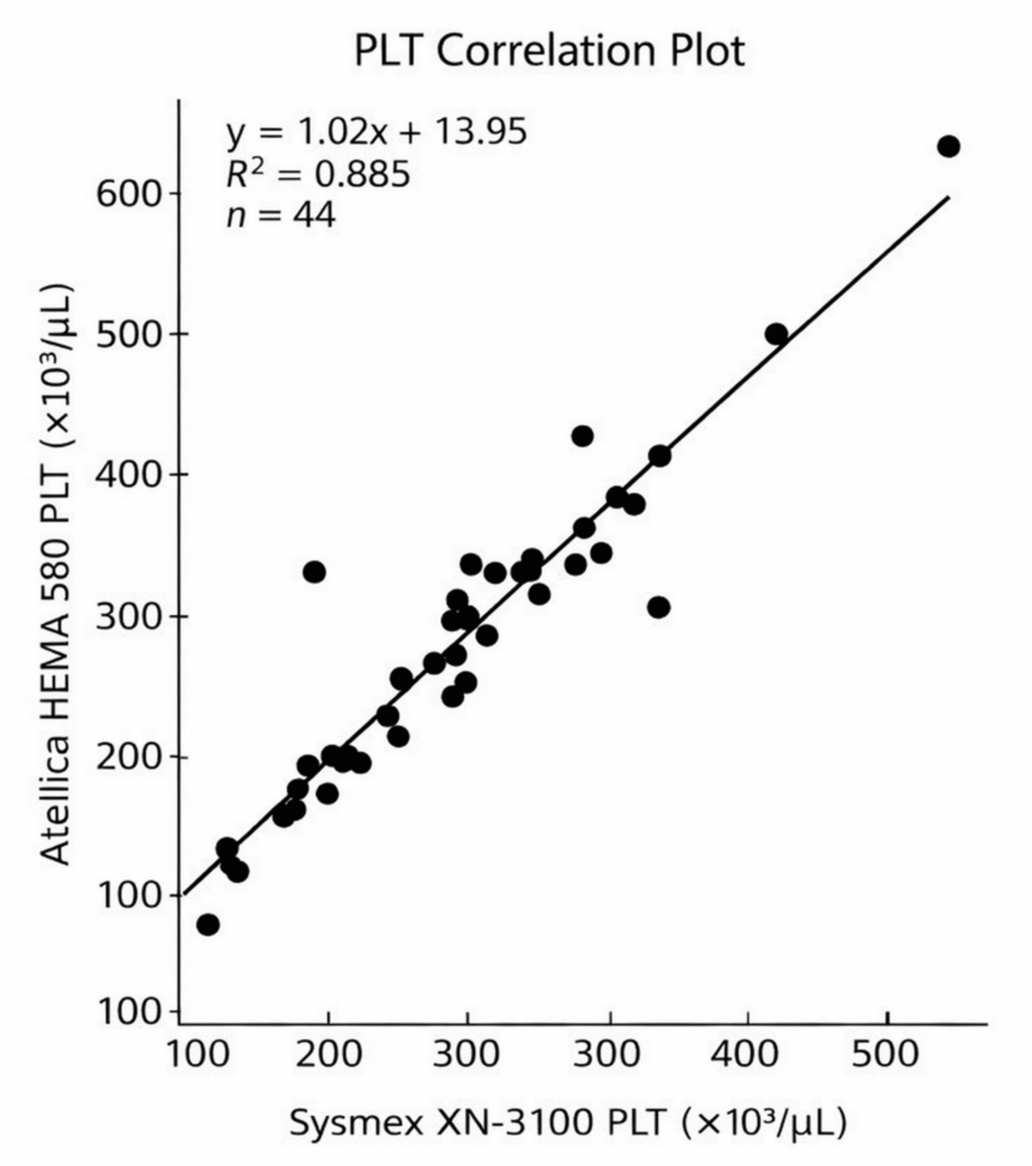
Method comparison of platelet (PLT) counts between Atellica HEMA 580 and Sysmex XN-3100 Scatter plot showing platelet counts measured on the Atellica HEMA 580 plotted against corresponding measurements from the Sysmex XN-3100 for 44 paired clinical samples. The solid line represents the line of best fit derived from ordinary least squares regression. The regression equation, coefficient of determination (R^2^), and sample size (n) are displayed within the plot. Platelet counts are expressed as ×10^9^/L. Deming regression was used to account for analytical error in both measurement procedures. Correlation is presented for method comparison purposes and should not be interpreted as analytical interchangeability without bias assessment.

**Figure 2:**
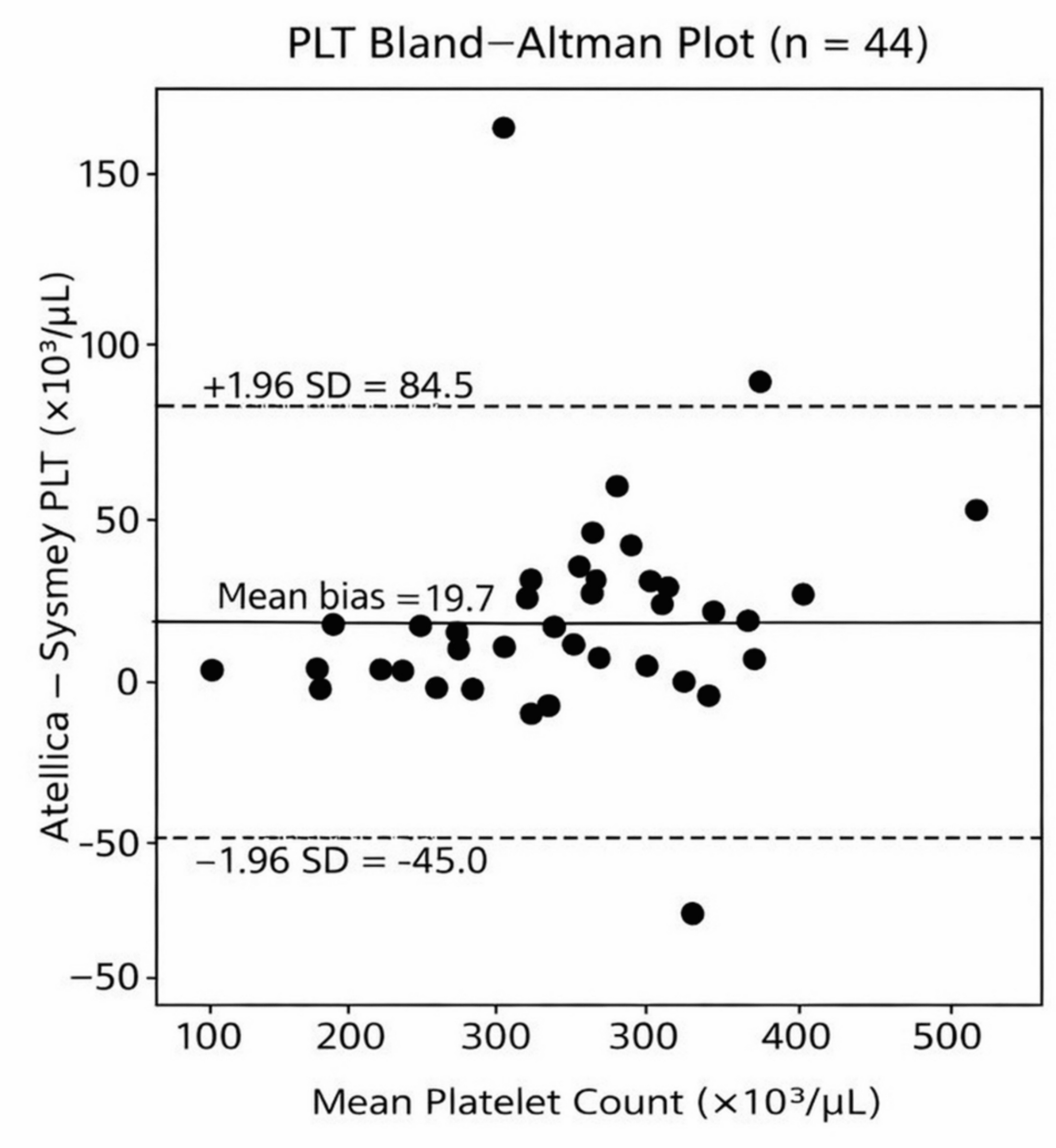
Bland-Altman analysis of platelet (PLT) count agreement between Atellica HEMA 580 and Sysmex XN-3100 Bland-Altman plot illustrating the difference in platelet counts (Atellica HEMA 580 minus Sysmex XN-3100) plotted against the mean platelet count for 44 paired samples. The solid horizontal line indicates the mean bias, while dashed lines represent the 95% limits of agreement (±1.96 standard deviations). Platelet counts are expressed as ×10^3^/µL. Limits of agreement were calculated as mean bias ±1.96 SD. The observed bias remained within ranges not expected to influence routine clinical platelet transfusion decision thresholds.

Strong analytical agreement was observed between the Atellica HEMA 580 and the Sysmex XN-3100 across WBC, RBC, hemoglobin, and platelet measurements based on 44 paired samples. Deming regression demonstrated a slope of 0.95 (95% CI, 0.91-0.99), indicating a modest negative proportional bias consistent with known methodological differences between impedance-based and optical platelet counting systems. Passing-Bablok regression confirmed absence of significant non-linearity or constant bias (Table 3 and Figures 1-2).

**Table 3:** Method comparison between Atellica HEMA 580 and Sysmex XN-3100 Method comparison was performed according to CLSI EP09-A3 using Deming and Passing-Bablok regression. Ninety-five percent confidence intervals (95% CI) are shown for regression parameters. CLSI: Clinical and Laboratory Standards Institute

Parameter	n	Deming slope (95% CI)	Deming intercept	Passing-Bablok slope	Mean bias
WBC (×10^9^/L)	44	1.02 (0.99-1.05)	-0.12	1.01	-0.04
RBC (×10^12^/L)	44	0.97 (0.94-1.01)	0.03	0.98	+0.01
Hemoglobin (g/dL)	44	1.01 (0.98-1.04)	-0.05	1.00	-0.10
Platelets (×10^9^/L)	44	0.95 (0.91-0.99)	+13.9	0.96	+19.7

Bland-Altman analysis demonstrated small and clinically negligible mean bias, with more than 95% of observations falling within limits of agreement and no evidence of concentration-dependent bias across the analytical range.

## Discussion

This CLSI-guided analytical validation demonstrates that the Atellica HEMA 580 delivers analytical performance characteristics consistent with current expectations for high-throughput automated hematology analyzers [3-6]. Across all evaluated domains - precision, linearity, method agreement, and carryover - the analyzer showed reproducible analytical performance comparable to the widely adopted Sysmex XN-3100 [9-11].

Precision is a cornerstone of CBC reliability, particularly in clinical scenarios requiring serial monitoring such as chemotherapy-associated cytopenias, infection follow-up, and transfusion decision-making. The very low within-run CVs observed for WBC, RBC, and hemoglobin minimize analytical noise and enhance confidence in detecting clinically meaningful trends over time. Platelet precision, while inherently more variable due to biological heterogeneity and methodological factors, remained within accepted analytical targets and aligns with published experience for impedance-based platelet enumeration systems [15].

Linearity findings further reinforce the quantitative robustness of the analyzer. Accurate performance across a wide analytical range, including values corresponding to severe anemia and erythrocytosis, is essential, as analytical inaccuracies at extremes may disproportionately influence diagnostic classification and therapeutic decisions [5]. The excellent proportionality demonstrated for both RBC and hemoglobin measurements confirms reliable analytical response across clinically relevant decision levels.

From a clinical perspective, the small analytical differences observed between the Atellica HEMA 580 and the Sysmex XN-3100 are unlikely to influence patient management when interpreted relative to established decision thresholds [17]. For hemoglobin, the observed mean bias and narrow confidence intervals remain well below commonly applied transfusion triggers. Similarly, minimal bias for WBC does not approach thresholds used for neutropenia grading or infection risk stratification. Platelet differences, although demonstrating modest proportional bias, remained within ranges unlikely to alter transfusion decisions [18].

Analytical harmonization between hematology platforms is increasingly important in laboratories operating mixed analyzer fleets or undergoing platform transitions [19]. The close agreement observed in this study - supported by regression slopes near unity and Bland-Altman limits within clinically acceptable ranges - supports continuity of longitudinal patient data without the need for correction factors [16].

Negligible carryover confirms effective fluidic system design and supports safe high-throughput operation in modern automated laboratory environments. This is particularly relevant for laboratories handling emergency, oncology, and critical care workloads. Limitations include the relatively modest sample size used for method comparison, the single-center design, the absence of differential leukocyte and morphologic assessment, and underrepresentation of pediatric and extreme pathological samples. Consequently, extrapolation of these findings to pediatric populations, uncommon hematological disorders, and specimens with extreme analytical values should be undertaken with caution.

## Conclusions

The Atellica HEMA 580 met CLSI analytical validation criteria and demonstrated strong analytical agreement with the Sysmex XN platform for the evaluated CBC parameters. Its high precision, excellent linearity, minimal analytical bias, and negligible carryover support routine clinical laboratory implementation within the scope of the analytical characteristics and study population assessed in this study.
